# A project co-created with the community to mitigate loneliness in midlife women

**DOI:** 10.3389/fpubh.2024.1425641

**Published:** 2024-09-11

**Authors:** Nadia Corsini, Fiona Dorman, Jodie Scott, Amanda Wright, Deborah Turnbull, Carmel Williams, Deborah Bates, Bernadette Reading, Hayley Everuss, Fanke Peng, Rachael Pearse, Marion Eckert

**Affiliations:** ^1^Co-Lab, Rosemary Bryant AO Research Centre, Clinical and Health Sciences, University of South Australia, Adelaide, SA, Australia; ^2^Premier’s Council for Women, Adelaide, SA, Australia; ^3^School of Psychology, The University of Adelaide, Adelaide, SA, Australia; ^4^School of Public Health, The University of Adelaide, Adelaide, SA, Australia; ^5^Centre for Health in All Policies Research Translation, Health Translation SA, SAHMRI, Adelaide, SA, Australia; ^6^The Hut Community Centre, Aldgate, SA, Australia; ^7^UniSA Creative, University of South Australia, Adelaide, SA, Australia; ^8^Uniting Communities, Adelaide, SA, Australia

**Keywords:** co-creation, loneliness, women, community initiatives, funding

## Abstract

This paper describes how a team of researchers, policy stakeholders and community members came together to co-create prevention-oriented and community-informed solutions to address loneliness in women—The Loneliness Project. Our aim is to encourage community partnerships and collective effort to address public health approaches to loneliness by developing a shared understanding of the issue from multiple perspectives and through the co-creation process, highlighting the key factors for co-creating a funding application for a community demonstration project.

## 1 Introduction

Participatory action research (PAR) is a research philosophy that includes the participation of community members as co-researchers to enable social change (1, 2). It aims to generate practical knowledge around issues of concern for the community and is particularly suited to promoting personal and social change (2). Key principles include respect for the knowledge of participants, mutual learning among them, recognition of the needs, and taking action for, marginalized people. In short, it is social research for social change (2).

Participatory action research encompasses the methods of co-creation, co-design, and co-production (2). Co-creation is an overarching construct that encompasses co-design and co-production. Co-design refers to the active collaboration of stakeholders to design solutions to a pre-specified problem, while co-production describes implementing solutions, where both the problem and solution were previously agreed—with an emphasis on making best use of existing resources (1). In comparison with the other two methods, co-creation refers to the collaborative approach of creative problem-solving between diverse stakeholders across *all* project stages (1). It begins with determining and defining the problem and runs through to the final stages of a project. It was devised as an approach to address complex public health issues that are considered “wicked” problems (3). Co-creation promotes the creation of value—either psychological, economic, or a social good. For this reason, co-creation is becoming increasingly important to help justify research costs to government and other funders, because it helps to direct resources to problems that matter to the community and deliver solutions that are policy and practice relevant and therefore more likely to be taken up and have impact. It allows research priorities to be led by the experiences of people who stand to benefit from the solutions, utilizing their lived experience and capitalizing on their ideas and energy for change, especially for complex challenges where research evidence is incomplete or requires contextualizing to a local community. Co-creation is an iterative process in which all relevant stakeholders contribute to solutions that may be novel or involve re-purposing what already exists. Many researchers will be familiar with co-creation methods that are used during a project or to prioritize and implement the most attainable research strategies. Co-creation can also be applied to describe the way in which diverse stakeholders work together in the very early stages to develop initial project ideas that can be pitched to funding bodies. It is this latter application of co-creation that is described in this paper.

Loneliness is a global epidemic affecting a range of ages and demographics. It is a subjective feeling that is activated when the frequency or quality of social connection a person is experiencing does not fulfill their needs (4). As described in the United States Surgeon General’s report (5), loneliness has a negative impact on population and community health, and strategies are needed at the individual, community, and policy level to support a connected community. Despite significant gaps, there is a wide range of evidence to inform solutions for loneliness. There have been efforts to synthesize the evidence on effective interventions (6–10), that combined with health promotion principles and social marketing theory, can inform public health whole-of-community approaches. Existing evidence can be considered together with what matters most to the community to design locally relevant strategies, with careful evaluation to monitor the effectiveness of innovations that are established.

There are examples of leadership globally and by governments to address loneliness at scale. The World Health Organization is supporting the implementation of the United Nations Decade of Healthy Aging (2021–2030) including publication of an online evidence gap map to assist with local solutions and support wider policy. The United Kingdom has developed and continues to implement their national loneliness strategy, with a dedicated loneliness minister. Japan has followed with a national strategy and minister. While national strategies can be important enablers, there is work that could be initiated by the collective efforts of local policy leaders, researchers, community organizations, and the public, but it can be difficult to visualize or fund initiatives to inform public policy and contribute to global efforts to address loneliness.

As many new initiatives to mitigate loneliness will require resources, teams of diverse stakeholders will need to come together to develop applications for funding. There are very few examples in the academic literature promoting the approaches and methods used by communities, researchers, and policy makers to work collaboratively on solutions that can be pitched to funding bodies. The Loneliness Project is an example of a diverse team of stakeholders co-creating a funding plan, a demonstration project within a single community center, and a policy-relevant scale-up plan.

The target population for The Loneliness Project is women in the middle years (40–65 years of age). Many studies show that women express feeling all loneliness indicators at higher rates than men (11, 12). Although loneliness can affect people of all ages, epidemiological data suggest there is an upward trajectory of loneliness in the middle years where it then stabilizes before peaking again in older age (11), but there is very little focus on this population group in the intervention literature. The middle years are associated with work and career transitions, relationship breakdowns, financial burden, and unpaid caring responsibilities for children and parents, as well as biological changes and societal norms that lead to more domestic work responsibilities (11). These can increase the chances of a person finding themselves disconnected. In addition, within our South Australian population, those aged 50–54 report high levels of loneliness—the second-highest age group by prevalence (13). Functional factors (such as health, or perceptions of social relationship quality) account more strongly for individual differences in loneliness than structural factors (such as social roles, networks and social activity) (14). At the same time, structural factors must be in place to support functional factors. In addition, older adults often fail to navigate their social lives around losses of health or social relationships (14)—hence the importance of strengthening social connections in midlife. By focusing on the middle years, the current project aims to strengthen connections that can help to prevent loneliness in the later years.

Inspired by the United States Surgeon General’s report (5), our project takes a public health approach by focusing on how loneliness can be mitigated among midlife women by addressing resources within one’s local neighborhood, via community centers. Supported by a national network (The Australian Neighborhood Houses and Centres Association), community centers are organizations that engage people, build community relationships, and have established connections with business, government, service providers and community leaders. The funding arrangements for community centers include a mix of funding from state governments, local councils, philanthropic sources, fundraising, and volunteer input. The focus on community centers is based on the premise of potential for wide-spread implementation. With over 1,000 centers in Australia (15), the project leverages the capacity of community centers to engage with midlife women to mitigate loneliness in later years.

In this paper we present a set of activities undertaken over 6 months that led to a successful grant application underpinned by a co-created topic, highlighting the challenges and factors of successful creative collaboration between diverse stakeholders.

## 2 Context

The current project is co-led by The Hut Community Centre, an independent center based in Aldgate in the Adelaide Hills. Aldgate has a population of approximately 3,500 people, over a third of are aged between 40 and 65 years of age, and Australian, English, Scottish, Irish and German are the top 5 ancestry groups (16). The Hut Community Centre has a comprehensive understanding of the population across the Adelaide Hills and the potential barriers to community engagement. These include a high number of small communities within the larger community, wide geographical area, physical barriers including major freeways separating localities, increased internet usage and working from home, and rising cost of living affecting people’s behavior including driving.

Funding for the Loneliness Project was obtained through an invitational process via the South Australian chapter of the national Women’s Health Research Translation and Impact Network (funded via the Medical Research Future Fund). Each state chapter invited one team of researchers to make an application based on issues identified by the national Women’s Health Policy (17), across one of five priority areas—maternal, sexual and reproductive health; healthy aging; chronic conditions and preventive health; and mental health. The topic identified by the South Australian team was healthy aging. Funding recipients were required to demonstrate meaningful collaboration between academic and consumer/community partners on definition of the problem, solutions, implementation, evaluation, and scale-up. This collaborative requirement meant than a co-creation guiding principle was essential across the project, including orienting to a topic within this priority area. We were given 6 months to develop a grant application to fund the delivery of the proposed project, which was to be delivered over a 2-year period. This 6-month period was supported by seed funding of AU$40,000.

**Figure 1:**
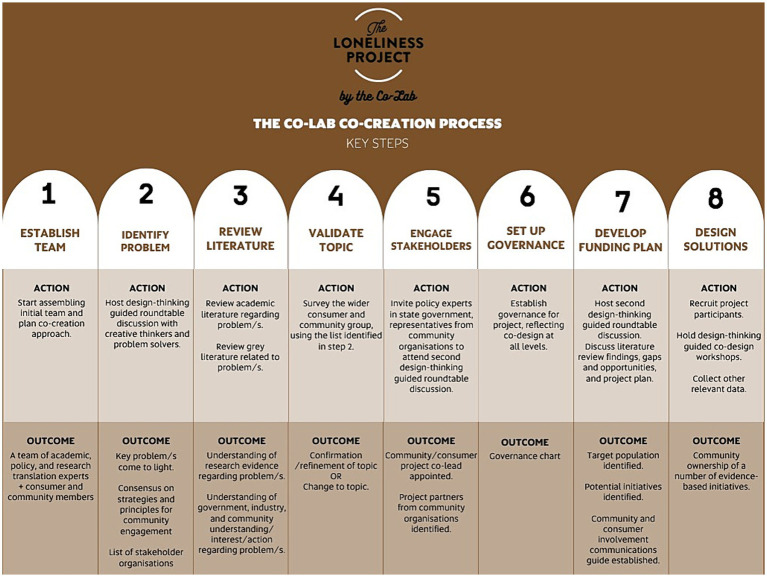
The Co-Lab co-creation process for The Loneliness Project.

The next section describes the iterative steps that facilitated the evolution of the co-creation funding plan over 6 months (Figure 1, steps 1–7).

## 3 Co-creation process key steps

Our co-creation process had seven main steps that are summarized in Figure 1 and elaborated on in the text. Step 8 includes the first 10 months of project delivery phase and completes the picture of activities to date. Step 8 is described later in the paper.

### 3.1 Step 1: establish the team (October 2022)

The aim of step 1 was to establish a team of 10 investigators for the grant application including five with academic, policy, or research translation expertise and five investigators from community organizations. The researchers were chosen for their expertise across women’s and public health topics and diversity of professional experience in key areas of policy, co-design approaches and knowledge translation—or identified through previous research collaborations. As we did not have community partners at this stage, our immediate challenge was to identify these project partners, which was addressed in step 2. The team received guidance and strategic advice from Health Translation SA, a National Health and Medical Research Council accredited research translation center in Australia. Much of this work was in-kind, with only two roles formally funded under the seed funding, and later, grant funding. Clarity of roles evolved along with the project, with groups and roles later defined under a Governance “Terms of Reference” agreement.

### 3.2 Step 2: develop engagement strategy to identify community partners (roundtable 1, December 2022)

The aim of step 2 was to develop the community engagement strategy to identify community partners. To start this process, we looked to our existing networks to identify women leaders in their fields (including public health policy, politics, journalism, business, and philanthropy) who were creative thinkers and problem solvers with collective broad experience. The women were invited to a “roundtable” discussion, along with community representation from two women. We engaged a design thinking expert (Author FP), who joined the project team, to help facilitate a 2-h roundtable discussion using a hybrid in-person/online format. All discussion participants were women in midlife (40–65 years), with the exception of two who were in their 30s.

The workshop posed an overarching question: How do we take the theme of healthy aging to the community, in a way that will engage, provoke questions, and therefore enable us to co-produce a research project that addresses an issue of high significance to women in Australia?

Participants workshopped three main topics:

WHAT: How would you describe healthy aging? Now combine the words Healthy Aging and Woman – what springs to mind?
WHO: Who must be included in the conversation about healthy aging for women? How would you start a conversation about healthy aging for women?
HOW: What should be the process for the priority setting discussions? What language/visuals/strategies would you use? What follow-up activities should be undertaken? What are potential stereotypes, concerns, or other issues to be mindful of? What is your main takeaway from today’s workshop?

Roundtable 1 aimed to generate ideas on the *process* of identifying community partners for the grant and engaging women in a discussion of healthy aging. We did not expect a topic to emerge at this stage. However, it was during this initial discussion that the topic of loneliness was first raised. There was extensive discussion about the challenges of healthy aging in midlife. Within this context, one discussion centered on the need for connection as a basic human drive. An observation followed that reflected on the COVID-19 lockdowns, during which one participant speculated that presentation to health care services was motivated as much by the need for interaction as the need to address the health issue. In the discussion about *process*, it was suggested that the key to engaging women about healthy aging could start with a question—What are women putting last? With the seed planted about social connection, healthy aging in the midlife, and putting oneself last, a key idea began to seed by the end of Roundtable 1—Are midlife women putting their social health last?

The workshop resulted in consensus on strategies and principles for community engagement and list of stakeholder organizations to be consulted.

Participants were invited to participate in a second roundtable.

### 3.3 Step 3: literature review (December 2022–February 2023)

In response to the discussion about social health in Roundtable 1, the researchers examined the literature on loneliness using a rapid targeted review process that sought to understand if loneliness was a priority for women and organizations interested in women’s health.

The scope of the literature review was targeted to include epidemiological trends for loneliness across the lifespan, drivers of loneliness, how loneliness spreads, and system-level strategies for addressing loneliness. We also confirmed that addressing loneliness was a federal and state government policy priority. It became clear that intervention studies related to midlife women were scant.

### 3.4 Step 4: online survey, validation of topic (February 2023)

An online survey was created to validate the topic and promoted through LinkedIn to organizations identified in Roundtable 1. The 28 responses to our survey validated our impression that loneliness was a priority for a range of organizations interested in women’s health. The survey was clearly framed as being relevant to all women across the life-course, hence not restricted to loneliness in midlife.

### 3.5 Step 5: stakeholder engagement (February–March 2023)

To ensure that our thinking and planning would be policy-relevant, we reached out to policy stakeholders in state government and community organizations with whom our team had connections. These experts were invited to take part in a second roundtable, and subsequently joined the project team.

We approached one of the participants from Roundtable 1 to be the Co-Lead for the project (FD), representing the community viewpoint for the grant. We scoped possible project partners who might be interested in developing a demonstration project, prioritizing organizations with whom we had existing relationships as we reasoned it would be difficult to build a new relationship within the grant timeframes. One of these organizations was The Hut Community Centre, who agreed to join the team and became the partner organization for the project.

### 3.6 Step 6: governance (March–April 2023)

**Figure 2:**
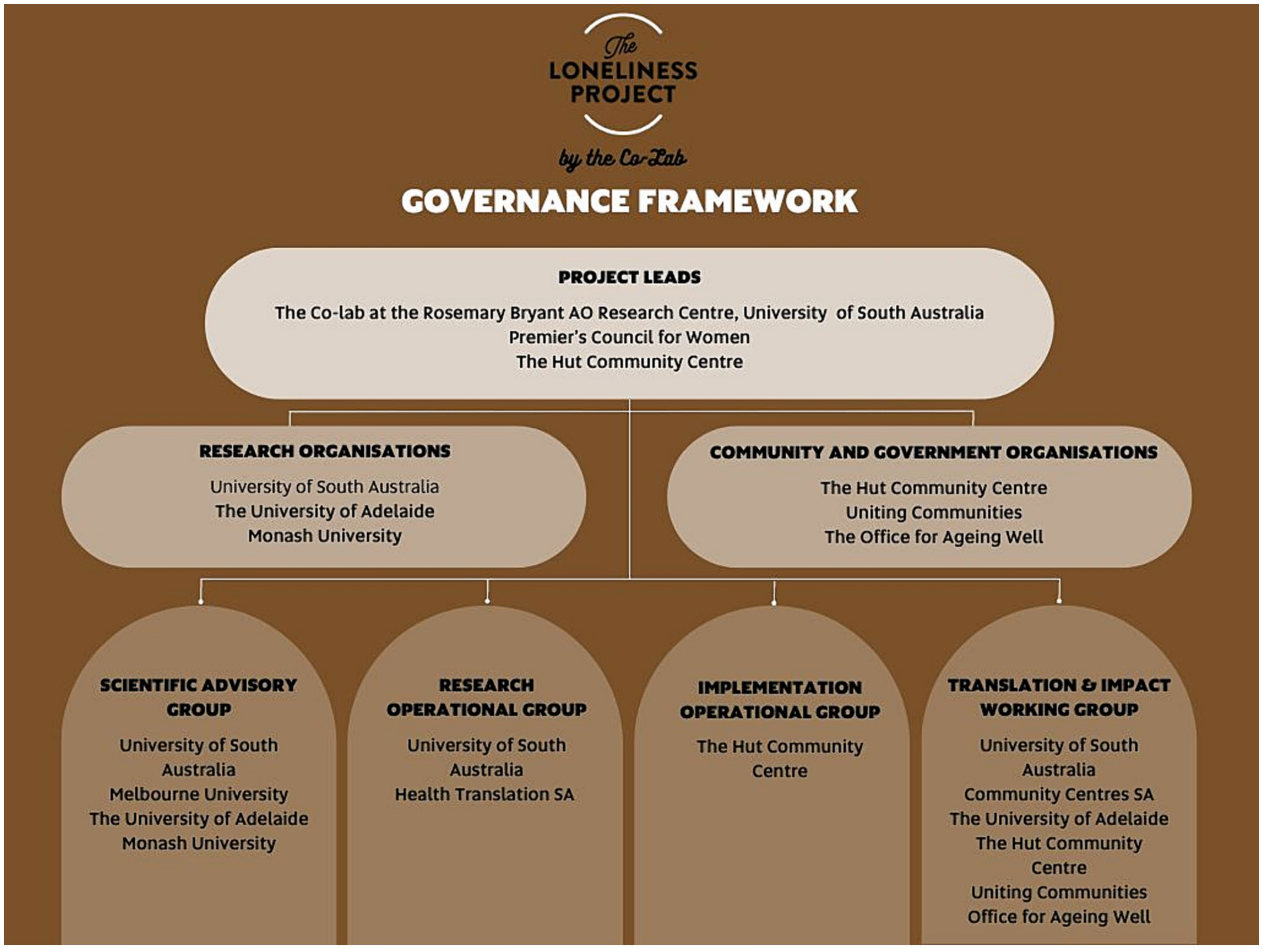
The Loneliness Project governance structure.

The governance structure for the project is represented in Figure 2. The leadership is shared by academic and community partners. The working groups are organized to promote scientific and methodological excellence (Scientific Advisory) and to embed a strategy to for translation and impact from the beginning (Translation and Impact). The operational groups were operating as one group at the time of writing, due to the close alignment of activities leading up to the community co-design workshops, which is described later in the section on project status.

### 3.7 Step 7: establish grant application plan (roundtable 2, March–April 2023)

The aim of Roundtable 2 was to establish the grant application plan. Leading up to this, weekly discussions were held with all team members about the literature review findings, gaps and opportunities, and a broad plan for the grant was sketched out over a 2-month period. Participants included most of the women from Roundtable 1, a policy leader, a community organization representative, and community center staff.

The workshop presented research evidence to support decisions regarding three overarching questions:

- Who is the target age group for our project?
  We presented the evidence about trends in loneliness and how midlife is a key period in an upward trajectory of loneliness. We argued that focusing on midlife offered potential as a preventive measure to increase social health prior to older age. It was evident that little attention is given to loneliness in midlife in the research literature and the popular media. Midlife women are under-represented at community centers.
- Could we use a podcast series to raise awareness about loneliness and support community connections?
  The gray literature review found that loneliness had been explored in several podcast series. We therefore took a close look at podcasts, an emerging infotainment trend among midlife women and a medium that can have an influence on changing attitudes, cognitions, and behavior across all levels of the social-ecological model (18). In addition, aspects of podcast listening such as parasocial relationships and social engagement are related to positive outcomes (19). The team explored the idea of a podcast series as a tool to raise awareness about loneliness, and help de-stigmatize and change thinking around loneliness, packaged in a friendly and entertaining format. We found examples of podcasts that created virtual communities of listeners with common interests, provided evidence-based information and a forum to share stories, shared information from experts and health professionals, and initiated calls-to-action on topics that are challenging. There were some podcasts about loneliness but none that focused on both our target audience and on building community connections—noting that the goal is to enable community centers to reach and connect with a new audience in midlife women.
- How should we talk about loneliness to avoid potential stigma?
  The issue of stigma was raised often in the literature reviewed. We therefore discussed whether the term loneliness should be used at all in outward facing promotion of the activities developed for the project. And if not, what terms or phrases might resonate instead. Discussion about stigma centered around how to get people to talk about loneliness, and how to market our initiatives to the community. Other initiatives have often taken one of two approaches: own the term “loneliness” as a method of de-stigmatizing or use the term “social connection” as a proxy to address loneliness. No consensus was reached.

In Roundtable 2, several key project decisions were reached (target population: midlife women; podcast: potential as a communication strategy) and the policy priorities were established (to strengthen the capacity of community centers). Further strategies were discussed about the types of innovations that could be delivered via community centers. It was agreed that the *specific* innovations would need to be informed by women in the community, and that further consultation would be needed during the project delivery.

The project plan was finalized over the next month, with submission of the grant being the final step of the 6-month process. The team received funding for a 2-year project with funds being allocated for both the research and delivery of the project. The next section outlines our reflections on the process.

## 4 Discussion

This paper describes the way in which a co-creation process focused on healthy aging in women enabled a team with varied backgrounds to come together to develop a funding application to deliver a project to mitigate loneliness. Below are some key reflections about the co-creation activities from varied perspectives: researchers, community organization staff, women from the community, and policy stakeholders. The purpose of these reflections is to share and disseminate our approach so that other groups can seek funding to support community projects to mitigate loneliness. We emphasize some challenges that might occur, reveal some lessons that we learned along the way, and highlight what we believe to be key factors for success in developing a funding application.

### 4.1 Researchers

A challenge for researchers in co-creation projects is to be comfortable with facilitating a process that encourages equal participation from all stakeholders, presents the research in a way that is accessible to the stakeholders, and encourages innovative and creative thinking. Researchers will need be comfortable with problem solving and being inventive rather than following a predictable method. In practice, it was difficult for the research team, who were ultimately responsible for writing the grant, to avoid dominating the narrative with academic arguments. A factor for success was being aware of this bias and aiming for a partnership of equals. This included recognizing the strengths that each set of experiences brought to the project and the seed funding was used to provide project support to all parties, not just the research team.

A key feature of the co-creation process is bidirectional and transparent communication (1), a factor that was held in high consideration across the process. To negate any potential power imbalances, our governance structure included project co-leads from academic, consumer and community sectors. In addition, our consumer representative co-lead brought an independent perspective to the project. In accordance with mechanisms of action in “co” approaches, engaging people as equal partners and valuing all forms of knowledge are key to addressing power differentials (20).

In addition, the research team created a safe environment where ideas could be shared, challenged, and debated. This opened up space for the other partners in the project to voice their thoughts, ideas and concerns, helping to generate a thoughtful, evidenced-based research project proposal to a complex issue. A safe co-creation environment—is essential to build trust across the diverse partners engaged in the process (1). An action that helped to promote this environment included inviting reflection and feedback from the group on the quality of the partnership during operational meetings, which enabled voicing and resolutions of concerns at early stages.

Due to the complexities of projects with multiple stakeholders, the set-up time is important to formulate the topic/challenge/question and form the team. A major enabler of project success was the seed funding as it as it enabled sufficient resourcing to bring together a team with the relevant set of skills and experience to engage in conversations and problem clarification, and by doing so supported topic evolution and consolidation of a shared vision by the project team.

We recommend that the party initiating the collaboration dedicate a project manager for at least 1 day per week over 6 months to co-ordinate the activities required to co-create a funding application.

### 4.2 The Hut Community Centre

Working toward a partnership of equals is important in a co-creation project. The Hut Community Centre became involved leading up to Step 7. At this point, the academic researchers were driving the agenda more than the community members. It has taken time to reach a position where everyone’s contributions are equal. This includes time to understand and utilize the team members skills and interest to support project success.

A key factor for success is allowing room for a co-creation project to mature and develop over time. In the current project, this was achieved by not limiting the scope of the project to the initial roundtables but allowing this to progress (and ultimately improve) with the input provided by the local community. There was acceptance by all parties that not all problems needed to be solved straight away. Some solutions can take time and room to think and consider. Funding to support staff resources at the community center was imperative to the effectiveness of this process, as it enabled a community development officer to work alongside an academic research associate during the project delivery phase.

We recommend that community organizations are adequately funded in the project budget by funding position(s) as well as cost associated with project delivery, including funding to engage with the community and reimburse community members.

### 4.3 Community members

It is vital to involve the community members in the decision making. Community initiatives will be more effective when the community members who will benefit have been involved in its creation, are highly engaged with the topic, and believe they will make a difference. Community engagement can typically be challenging. There was no time for engagement with The Hut Community Centre volunteers and visitors during the initial 6-month co-creation phase. This could be viewed as a major omission in defining the scope of the project, with the associated risk that the project issue may not resonate with the community broadly. A call for community support, albeit brief, may have validated the topic initiation process. It is acknowledged that community members will hold a vital role in the next phase of the project—co-design solutions, for which the grant funds have been allocated.

We recommend that the project team consider the skills and expertise needed for the project to determine whether individual community members or community organizations, or a combination, make up the project team. Depending on the project and timeline, the perspectives of individual women from the community where a specific initiative will take place could be very relevant and it may be appropriate to include one or more community members in a project team.

### 4.4 Policy stakeholders

One of the crucial ways that research can be applied practically, is to ensure it is supporting effective policy outcomes. Importantly, policy input should start early to ensure the research questions and project activities are linked to questions and outcomes that are relevant to developing effective policy. A key factor of success for the establishment of this co-creation project is the plan to replicate the outcomes/approaches that are effective into other community centers around Australia. Part of this process involves gathering compelling data to determine which elements of the solution are key ingredients to success and sharing the know-how for others to apply these elements in ways that suit their particular setting.

We recommend incorporating advice from policy stakeholders from the outset, to ensure that the project is gathering the information/resources needed to translate the research into practice within the setting of interest to mitigate loneliness.

### 4.5 Limitations

This paper describes the processes our team used to co-create solutions to address loneliness in women. We believe the approach is likely to work for teams in many different contexts. A limitation of our project relates to cultural diversity as our setting limits the reach to women from minority cultural groups in Australia, of which there are many. We acknowledge that the challenges, factors for success, and recommendations described in this paper assume that academic team will be the party initiating and driving the collaboration and funding application. We understand that this may not always be the case and recommend adapting the approach to the suit the context and needs of the stakeholder groups involved.

### 4.6 Project status—designing solutions with the community

We are currently 10 months into the project delivery phase and in the process of designing solutions to mitigate loneliness with the community (Figure 1, step 8). There was a very positive response to calls from The Hut Community Centre for women within the target age group and older to engage with designing solutions. Women could contribute in different ways: (1) by sharing their views anonymously in a survey, (2) being interviewed one-on-one about their loneliness experiences, and (3) taking part in three face-to-face workshops.

The workshops employed design-thinking methods, adapted from those developed by IDEO DesignKit online resources (21) and were developed and facilitated by members of the Operational Working Group. Run across three sessions with a week between each, the workshop took an iterative approach to idea generation. It followed an inspiration, ideation, and iteration methodology with the aim of building empathy and inspiring innovation. Our design challenge posed the question: How can we shape community centers to help prevent and address loneliness for women in midlife? Within this, the activities addressed a “how might we…” challenge, to allow divergent thinking (going wide to find insights), then convergent thinking (narrowing the focus to refine ideas).

At the time of writing, we had completed a series of community co-design workshops with 22 women in which the concept for four initiatives was developed. Following the workshop, 17 women volunteered to continue to develop and refine the initiatives with the community center. An evaluation of the co-design workshop and delivery of the initiatives is currently being developed. This activity occurred during the project delivery phase (i.e., Figure 1, step 8).

The four concepts to address loneliness were: (1) a community café, (2) a model for the delivery of group fun/educational activities for social connection, (3) a personal development program/course, and (4) a podcast—for conversations about loneliness, to de-stigmatize loneliness, role model positive social interactions, and to promote the work that community centers do to reduce loneliness and connect individuals to each other and their communities. At the time of publication, these initiatives were being developed for implementation.

While this project had a distinct focus and was aligned to a pressing issue within this single community in the Adelaide Hills, the model has potential to be used or adapted to address any social issue in a community context. The issues to be addressed may differ vastly across Australia or worldwide, and vary with the cultural and social diversity of a community, and across funding contexts.

## Data Availability

The datasets presented in this article are not readily available because the paper includes a small subset of evaluation data collected as part of the community co-design workshops. Data collected at the community co-design workshops will be published in subsequent papers, in which data availability will be described. Requests to access the datasets should be directed to nadia.corsini@unisa.edu.au.
